# From Preservation to Precision in Pediatric Dentistry: Evidence-Calibrated Viewpoint and Heuristic Framework for Silver Diamine Fluoride Guidance

**DOI:** 10.3390/children13050629

**Published:** 2026-04-30

**Authors:** Ziad D. Baghdadi

**Affiliations:** 1 Centre for Community Oral Health, College of Dentistry, University of Manitoba, Winnipeg, MB R3B 0L8, Canada; drziadeddin.albaghdadi@gmail.com or zalbaghdadi@atsu.edu; 2 Department of Preventive Dental Sciences Division of Pediatric Dentistry, College of Dentistry, University of Manitoba, Winnipeg, MB R3E 0W2, Canada; 3 Topsmiles Pediatric Dentistry & Orthodontics, Winnipeg, MB R2M 3A5, Canada; 4 Butterfly Dental Group, Winnipeg, MB R3Y 1P5, Canada

**Keywords:** silver diamine fluoride, pediatric dentistry, preservation-based care, precision dentistry, policy appraisal, guideline development, evidence certainty, caries arrest

## Abstract

Silver diamine fluoride (SDF) is a key preservation-based intervention in pediatric dentistry. It can arrest many cavitated lesions, reduce treatment burden, and expand access for children who cannot receive conventional restorative care. This viewpoint article offers a reasoned, heuristic framework for calibrating SDF guidance to the strength of the underlying evidence. It does not present a systematic review or formal policy standards. Foundational trials support the clinical usefulness of 38% SDF. The 2017 AAPD guidelines provided conditional recommendations based on low-quality evidence. The current challenge is no longer whether to endorse SDF but how to calibrate guidance on its implementation. Later studies addressing intervals and implementation often have open-label designs, small samples, single centers, or overlapping data sources. Mechanistic and microbiome studies support biological plausibility, but policy should not treat them as definitive evidence. We propose a hypothesis-generating framework that separates claims about the existence of an effect (for which there is stronger directional support) from claims about its optimal conditions (which remain more uncertain), highlights dataset overlap, and matches recommendation strength to study quality. The framework supplements GRADE and provides illustrative upgrade pathways. The goal is to preserve SDF access while making guidelines more transparent, credible, and precise.

## 1. Introduction

Preservation-based care is now central in pediatric dentistry. It reduces the operative burden, preserves tooth structure, and expands treatment options for children facing behavioral, geographic, financial, or medical barriers. Silver diamine fluoride (SDF) exemplifies this shift. It is quick to apply, requires no anesthesia or drilling, and helps stabilize the disease. SDF also preserves future restorative options. For clinicians serving high-risk children or those with limited access, this practical value is substantial [1,2].

However, preservation-based care is genuinely precise only if recommendations align with the certainty of supporting evidence. Clinicians need more than knowing *if* SDF can work; they must also know how confidently the field addresses preferred reapplication intervals, lesion arrest durability, the unit of analysis, and the extent to which findings apply across populations and systems. A convincing efficacy signal must be distinguished from the much harder task of policy calibration [3,4].

Calibration is a practical need, not just an academic issue. Guideline language affects referrals, reimbursement, training, consent, and planning. When implementation guidance overstates certainty, clinicians may wrongly believe that the best way to use SDF has already been proven. Making uncertainty explicit, however, makes minimally invasive care more credible and easier to improve [5].

This view offers a reasoned perspective for clinicians, guideline developers, educators, and researchers. The main argument is constructive: there is a real pediatric efficacy signal for 38% SDF that matters for policy. Nevertheless, guidance should separate claims with stronger directional support about caries arrest from less certain claims on implementation. The evidence-calibrated framework proposed here provides illustrative upgrade criteria and bridge rules for guideline panels.

## 2. Methods—Transparency and Scope

This manuscript presents a viewpoint-informed heuristic framework, not a systematic review, meta-analysis, or formal policy instrument. Its purpose is to synthesize existing evidence, guidelines, and critiques to propose a framework for SDF evidence calibration that readers can test, adapt, or reject.

**Selection transparency**

We did not conduct a formal systematic search. Instead, we used the following sources with explicit inclusion criteria:

- **All English-language clinical trials reporting 38% SDF for caries arrest in primary teeth** cited in the 2024 Cochrane review (n = 13 primary studies) [3]. Trials meeting these criteria were not excluded.
- All **AAPD guidelines, policies, and chairside resources** on SDF published between 2017 and 2025 [1,2,6].
- **Mechanistic and microbiome studies** cited in at least two major syntheses (Cochrane 2024 [3], AAPD 2017 [1], or Urquhart et al. 2019 [4]).
- **Methodological literature** on evidence certainty (GRADE) and implementation science [5,7].

A PRISMA-style flow diagram of the selection process is provided in Supplementary S1. Supplementary S2 provides a structured summary of all included studies, organized by evidence domain, with key characteristics (sample size, centers, masking, risk-of-bias summary) and a notation of source-independence status. Supplementary S3 provides the expanded source Independence Matrix. Supplementary S4 contains the risk-of-bias assessment details (Cochrane RoB 2 Domains).

For the five additional Cochrane trials not discussed individually in the main text, a grouped summary with basic descriptors is provided, along with a footnote explaining that detailed risk-of-bias scoring was not performed as they do not bear directly on the framework’s central claims.

### Limitations Acknowledged

This approach has inherent limitations: no formal search protocol, no duplicate screening, no quantitative synthesis, and no risk of selection bias. The framework is **hypothesis-generating**, not hypothesis-testing. Independent replication via a systematic search would be valuable.

## 3. Why Silver Diamine Fluoride Matters in Preservation-Based Pediatric Care

The clinical case of SDF is clear. Some young children with severe caries cannot cooperate with conventional treatment, lack access to specialists or resources, or face social and financial barriers. In these cases, SDF offers an evidence-based, minimally invasive option that can delay or reduce the need for extensive intervention, preserve tooth function, and enable stabilization, behavioral guidance, and risk reassessment [1,8,9,10,11].

Foundational trials across multiple settings show that SDF can arrest dental caries in primary teeth. The effect is not identical across all lesions, tooth types, or contexts, but it is generally favorable. Contemporary reviews, including the 2024 Cochrane review, support this signal and rate the certainty of evidence as **low** (GRADE) [3].

That combination—clinical usefulness alongside limited certainty—is important. Treatment may justify adoption even when not all implementation details are settled. In pragmatic pediatric care, this distinction is often necessary. The key challenge is not simply whether SDF has a place in the armamentarium but whether policy documents clearly distinguish between well-supported and provisional claims [1,3].

## 4. SDF in the Treatment Continuum: When Preservation Is Enough and When It Is Not

SDF is indicated for cavitated lesions without clinical signs of pulpal involvement. For teeth with irreversible pulpitis or necrosis, SDF is **not** a substitute for pulpectomy, extraction, or advanced restorative procedures for permanent teeth. As demonstrated in systematic analyses of endodontic instrumentation [12], technical precision is critical when transitioning from preservation to more invasive treatment. Similarly, for permanent teeth where preservation is possible despite advanced disease, techniques such as surgical extrusion [13] represent advanced restorative options that remind readers that “preservation” is a spectrum, not a binary state. The present framework focuses on SDF’s role in the early-to-mid part of that continuum.

## 5. Current Guideline Language: Appropriate Endorsement, Uneven Calibration

The 2017 AAPD guidelines are well-balanced. The GRADE approach was used, and 38% SDF was **conditionally recommended** for caries management of cavitated lesions in primary teeth. The guidelines clearly state that this approach is based on low-quality evidence [1]. This approach was methodologically restrained: benefits outweighed harms, but certainty was not overstated.

Since then, AAPD chairside materials have continued to support SDF in ongoing caries management, stressing follow-up, monitoring, and the possibility of repeated applications [2,6]. These clinical statements make sense, but the evidence standard for broad implementation claims is greater than that for general efficacy.

The main policy issue is **calibration, not endorsement**. General support for SDF makes sense. Stronger claims about optimal intervals, lasting arrest, or generalizability need more evidence. Without separating these claim types, readers might mistakenly assume that detailed guidance is also supported by broad efficacy, which is not accurate [3].

## 6. The Architecture of the Current Evidence Base

The SDF literature relevant to pediatric guidance can be organized into five distinct evidence domains. Notably, these domains differ significantly in terms of the weight they should carry in policy formulation.

**Foundational efficacy trials** provide the strongest anchor. Studies by Llodra and colleagues, Yee and colleagues, Zhi and colleagues, Fung and colleagues, and related trial programs demonstrated that SDF can arrest carious lesions in primary teeth and that concentration and periodicity matter [8,9,10,11]. These trials directly address the outcome that matters most to policy: whether lesions arrest under real pediatric conditions.

**Implementation and interval studies**, including North American trials comparing reapplication schedules, have moved the literature closer to the questions clinicians ask in practice. However, many studies have remained open-label, single-center, or modest in sample size [14,15]. They inform implementation but do not resolve it with high certainty.

**Patient-centered outcome studies** and **mechanistic studies** enrich the evidence ecosystem. Oral health-related quality-of-life analyses help determine whether preservation-based management is acceptable to families [16]. Microbiome and mechanistic studies contribute to biological plausibility and generate hypotheses about which lesions are more or less likely to respond [17]. Their primary value is explanatory or supportive, not policy-settling.

**Systematic reviews and guidelines** aggregate the literature but inherit the strengths and weaknesses of underlying studies. A well-conducted guideline cannot convert low-certainty primary evidence into high-certainty conclusions by simply synthesizing it [3,4].

Table 1 summarizes these five evidence domains, their typical examples, strengths, limitations, and—most importantly—the **appropriate policy weight** that evidence from that domain should carry.

## 7. Methodological Limitations of the Current Study

A recurring weakness in later supportive studies is incomplete protection against bias. Open-label designs are common, particularly in pragmatic or community-based trials where masking is difficult. This is understandable, and some implementation scientists argue that open-label pragmatic trials are fit for purpose for real-world questions. However, when the primary endpoint involves lesion hardness, color, or clinical arrest judgments that can be influenced by examiner expectations, confidence in precise regimen claims should be lower than confidence in the broader proposition that SDF can be effective [14,15].

A second limitation is sample size and center dependence. Later supportive studies often involve modest numbers of children, especially when subgroup analyses or mechanistic end points are involved. Small samples can still produce useful signals, but are less reliable for determining optimal intervals or supporting broad generalization. Single-center designs raise questions about transferability [14,15].

A third limitation concerns confounding and measurement precision. Diet, visible plaque, oral hygiene, and background fluoride exposure all influence lesion arrest. Studies that do not measure or control these factors may still be clinically informative, but their findings should not settle implementation policy [15,17].

A fourth limitation arises in mechanistic studies that rely on whole-mouth or non-site-specific sampling. Such designs weaken causal inference about what is happening at the treated lesion. Mechanistic studies support biological plausibility and hypothesis generation without carrying significant weight in policy-defining claims [17].

A fifth issue is **source independence**. In an emerging field, a single trial platform may yield several outputs: a main efficacy or interval paper, a quality-of-life analysis, and one or more mechanistic papers. This is legitimate. However, guideline panels and readers must avoid treating these outputs as equivalent to multiple independent confirmatory datasets. Without explicit de-duplication, the evidence base can appear broader and more mature than it actually is [15,16,17].

## 8. Evidence Inflation from Overlapping Trial Platforms: A Structural Concern

The risk of evidence inflation is best illustrated with a systematic map. Table 1 provides a source-independence matrix for major research platforms (the full matrix is in Supplementary S3).

The structural point is that no single research group is at fault. The risk emerges when later supportive papers are cited in guidelines, reviews, or training materials in a way that blurs the distinction between a valuable contribution and a confirmatory policy-grade trial. The solution is not to discount such studies but to classify them transparently according to what they can and cannot provide [3].

## 9. Evidence Variation by Lesion and Patient Characteristics

Confidence in SDF’s effectiveness is not uniform across all clinical scenarios. The following summary reflects the author’s interpretive synthesis of the available literature [1,2,3,6,8,9,10,11].

**Lesion-specific considerations**

- **Depth:** Several studies have shown that deeper lesions (into dentin) exhibit greater arrest rates than shallow enamel lesions. However, lesions approaching the pulp present an unclear risk-benefit trade-off; no high-quality evidence supports the use of SDF when pulpal involvement is suspected [10,11].
- **Location:** Proximal lesions are less studied than buccal/lingual lesions. Therefore, generalization to proximal surfaces requires caution [8,9].
- **Pulpal involvement:** SDF is **not indicated** for teeth with signs or symptoms of irreversible pulpitis or necrosis. This framework does not apply to such cases.

**Patient-specific considerations**

- **Age:** Most trials included children aged 2–6 years. The generalizability of these findings to older children with different caries patterns is uncertain [8,9,10,11,14,15].
- **Behavior status:** SDF is often used for uncooperative children, but evidence specific to this subgroup is limited [14].
- **Caries severity:** High-risk children with multiple lesions are well-represented; children with single lesions may have different response profiles [10,11].

## 10. Policy Options and Implications

Three broad policy options are available [1,3,5]:

- **Maintain current broad endorsement with minor wording changes.** This approach preserves continuity but risks allowing implementation claims to sound more certain than the evidence justifies.
- **A tiered evidence-calibrated model** (proposed here). Guidance would separate high-confidence efficacy statements from lower-confidence implementation statements, with explicit acknowledgment of the differences in certainty.
- **Reserve strong interval-specific wording for independent confirmatory evidence.** This approach avoids a strong preference for a particular interval unless supported by multicenter, methodologically rigorous, independently replicated trials. The trade-off is less immediate operational specificity.

For most pediatric dentistry systems, the second option is most balanced. It preserves access, recognizes clinical utility, and improves transparency without waiting for perfect evidence.

## 11. Proposed Considerations for Guideline Developers

The following are **proposed considerations** and normative suggestions based on principles of evidence transparency [5,7]. They are not formal policy standards. Different guideline panels may set different thresholds.

Consideration 1: Grade efficacy claims separately from implementation claims

The question “Does SDF arrest many lesions in primary teeth?” should not receive the same certainty rating as “What is the preferred reapplication interval for high-risk preschool children across diverse settings?”

*Implementation formulation:* Guideline documents should use separate certainty ratings for the claim “SDF can arrest caries” versus “SDF should be reapplied every X months.”

Consideration 2: Include a source-independence column in evidence tables

When multiple papers arise from a common registered trial platform or overlapping cohort, that relationship should be visible to panelists and readers.

*Implementation formulation:* Evidence tables should include a column labeled “Source independence status” with entries: “Independent cohorts”/“Overlapping cohorts—specify relationship”/“Unknown—unable to determine.”

Consideration 3: Use wording tiers in policy documents

Statements supported mainly by foundational efficacy trials may be written with greater confidence than statements resting on exploratory, supportive, or overlapping studies.

*Implementation formulation:* Use “SDF is effective for…” for Tier 1; “SDF may be considered for…” for Tier 2; “Research suggests that…” for Tier 3.

Consideration 4: Establish routine methodological expectations for studies intended to influence interval-specific policy

The following are desirable methodological features for studies that aim to inform interval-specific policy: examiner calibration and intra-examiner reliability reporting; masking strategy or centralized adjudication where feasible; explicit unit-of-analysis choices; handling of clustering by child; and measurement of key confounders (visible plaque, diet, oral hygiene).

*Implementation formulation:* Journals **may consider** developing a standardized checklist for SDF trials that includes these items, but this is not proposed as a mandatory requirement.

Consideration 5: Integrate mechanistic studies as complementary evidence rather than substitutes for robust clinical endpoint trials

Their role is to explain, stratify, and generate hypotheses, not to settle implementation policy alone.

*Implementation formulation:* Systematic reviews should code mechanistic studies as “explanatory only” and exclude them from primary evidence syntheses for interval recommendations.

Consideration 6: Make uncertainty visible in training materials and chairside resources

By distinguishing “what is established” from “what remains under active study,” such transparency builds trust.

*Implementation formulation:* Chairside resources should include a box labeled “What we do not know yet” alongside clinical guidance.

## 12. Evidence-Calibrated Hierarchy for SDF Guidance Statements

Table 2 operationalizes the framework by translating evidence domains into illustrative guidance language. The table organizes SDF-related claims into three **tiers**—broad efficacy (existence of effect), implementation (parameters of effect), and optimization/mechanism (explanatory)—each with wording that reflects the inferential strength the evidence can support. An upgrade pathway specifies how a claim could move from a lower tier to a higher tier as stronger evidence accumulates.

**Important clarification:** Both Tier 1 and Tier 2 currently rely on evidence that GRADE rates as *low certainty* [3]. This hierarchy reflects inferential strength for different *types* of claims, not different certainty ratings. The convergence of multiple heterogeneous trials in a favorable direction of effect supports a stronger inference about the *existence* of an effect than about its *optimal parameters* [3,8,9,10,11].

## 13. Worked Example: Applying the Framework to an Interval Claim

Table 3 contrasts appropriate wording (“may be considered; evidence is limited”) with inappropriate wording (“standard of care”), illustrating how the framework guides precise communication of evidence strength.

## 14. What This Framework Means for Clinicians—A Practical Summary

*Reasonably established (can inform clinical decisions now):*

- SDF (38%) can arrest many cavitated carious lesions in primary teeth [8,9,10,11].
- Its application is safe and well-tolerated in most children [1,2].
- SDF is a reasonable alternative when conventional restoration is unavailable or inadvisable (for lesions without pulpal involvement) [3,14].

*Remains uncertain (should not drive rigid policy):*

- The optimal reapplication interval (individualization is needed) [15].
- Arrest durability beyond 12–24 months [14,15].
- Which lesion type (depth or location) responds best [10,11].

*Should not yet be treated as settled:*

- Any single preferred interval across all populations [15].
- Mechanistic claims as policy-grade evidence [17].
- Comparative effectiveness versus specific restorative techniques without masked trials [14].

## 15. Implications for Practice, Education, Reimbursement, and Research

Reimbursement and service planning should explicitly distinguish SDF as an **interim stabilization tool** from SDF as a **long-term management strategy**. When payment policies or quality metrics treat SDF applications as a terminal endpoint, they risk incentivizing “bridge-to-nowhere” patterns. Therefore, calibrated guidance should encourage payers to link SDF coverage to documented recall plans and, where indicated, to subsequent definitive care [5]. These are plausible but untested proposals; pilot implementation would be needed before system-wide adoption.

## 16. Discussion

The most important conclusion is that **calibration, not opposition**, should guide the next phase of SDF policy development. The field does not need less preservation-based care. Rather, a more methodologically exacting translation of preservation-based evidence into policy is needed, preserving strong endorsement where warranted while avoiding rhetorical certainty in areas where evidence remains heterogeneous, overlapping, or vulnerable to bias [1,3,5].

Despite the limitations discussed, the SDF evidence base has several strengths: consistency of effect direction across heterogeneous settings, biological plausibility supported by mechanistic studies, and favorable safety profiles in thousands of treated children [8,9,10,11,17]. The goal of calibration is not to diminish these strengths but to ensure that claims match their evidentiary support.

### 16.1. Addressing the Central Tension: Efficacy Versus Implementation Dependence

A reader might object: “If the evidence for arrest is itself of low certainty (per Cochrane 2024 [3]), then your Tier 1 claims are also uncertain. You cannot claim high confidence in efficacy while simultaneously endorsing Cochrane’s low certainty rating.”

This objection is valid and requires a direct response. The Cochrane review rated the certainty of evidence as **low** for the outcome “caries arrest” across all comparisons [3]. The present review does not dispute that rating. However, “low certainty” in GRADE means that further research is likely to change the *estimate* of effect, not that the effect is absent or that the treatment should not be used [7]. The distinction we draw is not between “high-certainty efficacy” and “low-certainty implementation”. Rather, it is between:

- **Claims about the existence of a treatment effect** (SDF *can* arrest lesions under some conditions)—supported by multiple trials with consistent direction of effect, albeit with methodological limitations [8,9,10,11].
- **Claims about the optimal conditions for that effect** (specific intervals, population transferability)—supported by fewer studies, often with additional limitations (open-label design, single-center, confounding) [14,15].

Both sets of claims rest on low-certainty evidence in GRADE terms. However, the strength of the inference for the *existence* of an effect is greater than that for precise implementation parameters because the former is supported by convergence across heterogeneous settings, while the latter requires more demanding comparisons. The proposed framework operationalizes this difference.

### 16.2. Potential Objections and Responses

**Objection 1:** “Pragmatic trials are appropriately open-label. Demanding masking for implementation questions imposes artificial constraints that reduce external validity.”

**Response:** Open-label designs are most defensible when outcomes are objective (e.g., mortality, tooth extraction) or when blinding is truly impossible. The caries arrest assessment (lesion hardness, color, dentin texture) includes a subjective component. The appropriate middle ground is not to reject open-label studies but to discount their interval-specific conclusions relative to masked trials and to encourage centralized adjudication of outcomes using standardized photographs, where feasible [5,14,15].

**Objection 2:** “Clinicians need interval guidance now, not after perfect trials. Your framework might lead payers to withhold coverage for off-interval use, harming access.”

**Response:** This is a serious concern. The proposed framework explicitly avoids recommending against any particular interval. It recommends that guidance use conditional language (“may,” “can be considered”) rather than declarative certainty. Payers should **not** infer that evidence-calibrated language justifies the denial of coverage. The framework supports coverage for SDF without requiring a specific interval because the broad efficacy claim is sufficient to justify access [8,9,10,11].

**Objection 3:** “Your framework would reduce SDF uptake by introducing uncertainty that clinicians and families find confusing.”

**Response:** Evidence from risk communication research suggests that transparency about uncertainty does not reduce uptake of beneficial interventions when the bottom-line recommendation remains clear [5]. A parent can be told, “SDF is a good option for stopping cavities in many children. We do not know exactly how many applications work best for every child, but we will monitor your child’s response and adjust as needed.” This is both honest and clinically useful.

**Objection 4:** “You have not proposed a clear decision rule for when evidence moves from Tier 2 to Tier 1.”

**Response:** Table 2 now includes an illustrative upgrade pathway: at least 2 multicenter, masked trials with low risk of bias that show consistent interval-specific effects. This is a high bar—intentionally so—to prevent premature hardening of interval claims. Different guideline panels may set different thresholds.

### 16.3. When SDF Is Not Enough: Recognizing Treatment Limits

Clinicians must recognize when preservation-based approaches have reached their limits. For teeth with irreversible pulpitis or necrotic pulp, SDF is not an alternative to definitive endodontic treatment or extraction. In permanent teeth where preservation is possible despite advanced disease, techniques such as surgical extrusion [13] represent advanced restorative options. The present framework does not apply to such cases, and clinicians should avoid using SDF as a substitute for indicated definitive care.

### 16.4. Limitations of This Viewpoint

This narrative viewpoint has several limitations. A systematic search or meta-analysis was not conducted. Evidence classification reflects the author’s judgment rather than a formal consensus process. The proposed framework has not been tested in a real guideline development process. The case examples, while intended to be structural, may still be perceived as singling out specific research groups despite the inclusion of multiple examples. Finally, this viewpoint does not address all SDF policy questions, such as cost-effectiveness, variation in application technique, or use on permanent teeth.

## 17. Future Directions

A precision-oriented future for pediatric dentistry will depend not only on better diagnostics and tailored therapies but also on better evidence governance. The strength of recommendations should be mapped to methodological strength. Dataset overlap should be visible. Exploratory evidence should be labeled exploratory. Interval claims should be confirmed independently before they harden into operational dogma. When these principles are followed, preservation-based care does not become weaker; it becomes more credible, more adaptable, and more truly precise [3,5,7].

## Figures and Tables

**Table 1:** Evidence domains supporting pediatric SDF guidance and their appropriate policy weights.

Evidence Domain	Typical Examples	Main Strengths	Common Limitations	Appropriate Policy Weight
Foundational efficacy trials	Llodra 2005 [8]; Yee 2009 [9]; Zhi 2012 [10]; Fung 2016 [11]	Directly test lesion arrest in pediatric populations; repeated favorable signal across settings	Heterogeneity in protocols and follow-up; some older designs do not meet contemporary ideal trial standards	**High** relative weight for broad efficacy claims (existence of effect)
Implementation and interval studies	Cleary 2022 [14]; Schroth 2024 [15]	Closer to real clinical questions about timing and comparative management pathways	Often open-label, modest sample size, less robust for definitive interval preference	**Moderate** weight for conditional implementation guidance
Patient-centered outcomes	Sihra 2020 [16] and related quality-of-life analyses	Adds family-centered relevance and acceptability information	Frequently secondary analyses or underpowered for between-regimen conclusions	**Supportive** but not policy-defining
Mechanistic and microbiome studies	Manerkar 2025 [17] and related ecological analyses	Adds biological plausibility; may inform response hypotheses and future precision models	Often exploratory; non-site-specific sampling and confounding limit causal interpretation	**Complementary**; should not settle interval policy
Systematic reviews and guidelines	AAPD 2017 [1]; Cochrane 2024 [3]; Urquhart 2019 [4]	Aggregate multiple studies; provide quantitative summaries; GRADE ratings	Inherent limitations of primary studies; heterogeneity may obscure subgroup effects	**High** for characterizing overall evidence base; **low** for novel claims not in primary data
Source-independence matrix for major SDF research platforms (summary)				
Research platform	Component publications		Cohort overlap	Independence status
Hong Kong group	Zhi 2012 [10]; Fung 2016 [11]; related papers		Overlapping cohorts; same trial platform	**Overlapping**—not independent confirmations
Schroth group (Canada)	Schroth 2024 [15] (interval trial); Manerkar 2025 [17] (microbiome); Sihra 2020 [16] (QoL)		Same cohort; secondary analyses	**Overlapping**—multiple outputs from one trial
Cleary group (USA)	Cleary 2022 [14]		Single cohort; no derivative publications	**Single study**—no overlap issue
Llodra group (Cuba)	Llodra 2005 [8]		Independent cohort	**Independent**

**Table 2:** Evidence-calibrated hierarchy for SDF guidance statements with upgrade criteria.

Tier	Claim Type	Illustrative Wording	Minimum Evidentiary Threshold	Upgrade Pathway (Illustrative)
Tier 1	Broad efficacy (existence of effect)	“38% SDF is a valuable minimally invasive option for arresting many cavitated carious lesions in primary teeth…”	Multiple independent clinical trials showing consistent direction of effect, with at least two trials at low risk of bias (Cochrane RoB 2)	N/A (highest tier for this claim type)
Tier 2	Implementation (parameters of effect)	“Repeated application and follow-up may be needed to achieve or sustain arrest; exact interval should be individualized…”	At least one direct study plus external consistency, with explicit acknowledgment of design limitations	Requires independent replication in ≥2 multicenter, masked trials with low risk of bias *
Tier 3	Optimization and mechanism	“Microbiome shifts, lesion-level modifiers of response, and population-specific scheduling strategies remain under active study.”	Exploratory, mechanistic, single-center, or overlapping-cohort studies	Requires prospective confirmation in a Tier 2 or Tier 1 study design

* Different guideline panels may set different thresholds; this is an illustrative bar intended to prevent premature hardening of interval claims.

**Table 3:** Worked Example: Applying the Evidence Framework to an Interval Claim (6-Month SDF Reapplication for High-Risk Preschool Children).

Element	Example
**Claim to evaluate**	“SDF should be reapplied every 6 months for optimal caries arrest in high-risk preschool children.”
**Claim type**	Tier 2 (implementation)
**Evidence available**	One open-label, single-center trial (Schroth 2024 [15]; N = 120)
**Upgrade criteria met?**	No (requires ≥ 2 multicenter masked trials)
**Appropriate wording**	“Reapplication every 6 months *may be considered*; evidence is limited, and individualization is warranted.”
**Inappropriate wording**	“The standard of care is a 6-month reapplication.”

## Data Availability

The data presented in this study are available on request from the corresponding author. The data are not publicly available due to privacy and ethical reasons.

## Supplementary Materials

The following supporting information can be downloaded at: https://www.mdpi.com/article/10.3390/children13050629/s1, Supplementary S1: A PRISMA-style flow diagram of the selection process. Supplementary S2: A structured summary of all included studies, organized by evidence domain, with key characteristics (sample size, centers, masking, risk-of-bias summary) and a notation of source-independence status. Supplementary S3: The expanded source Independence Matrix. Supplementary S4: The risk-of-bias assessment details (Cochrane RoB 2 Domains).
