# Supplementary material for: From Preservation to Precision in Pediatric Dentistry: Evidence-Calibrated Viewpoint and Heuristic Framework for Silver Diamine Fluoride Guidance
Source: Children (Basel). 2026 Apr 30;13(5):629. doi: 10.3390/children13050629 (PMC13204953; doi:10.3390/children13050629)
Supplement: Supplementary file 1 [file children-13-00629-s001.zip › children-4273197-supplementary.pdf]

Supplementary Material

Supplementary S1 – PRISMA Flow Diagram

Selection of studies for the viewpoint framework

```
Records identified from Cochrane 2024 review (n = 13 clinical trials)
        ↓
Records screened (n = 13)
        ↓
Records excluded (n = 0) - all met inclusion criteria
        ↓
Full-text clinical trials assessed for eligibility (n = 13)
        ↓
Additional records from major syntheses for mechanistic studies:
- Cochrane 2024 cited mechanistic studies (n = 8 identified)
- AAPD 2017 cited mechanistic studies (n = 5 identified)
- Urquhart 2019 cited mechanistic studies (n = 4 identified)
- After deduplication: n = 9 unique mechanistic/microbiome studies
        ↓
Mechanistic studies screened against inclusion criteria:
- Cited in ≥2 major syntheses? (n = 5 met criterion)
- Cited in only 1 synthesis? (n = 4 excluded)
        ↓
Guidelines and policy documents:
- AAPD guidelines 2017, 2025 (n = 2)
- AAPD chairside resources (n = 2)
- Other major syntheses (Cochrane 2024, Urquhart 2019, Gao 2016) (n = 3)
        ↓
Methodological literature (GRADE, implementation science) (n = 6)
        ↓
Total included sources for framework development:
- Clinical trials: 13
- Mechanistic/microbiome studies: 5
- Guidelines/policy documents: 7
- Methodological references: 6
- **Total unique references informing framework: 31**
```

Supplementary S2 – List of All Included Studies with Key Characteristics

Clinical trials (foundational and implementation)

Study	Domain	Sample size (children)	Centers	Masking of outcome assessment	RoB 2 summary	Source independence
Llodra 2005	Foundational	~300 (cluster)	Multiple (Cuba)	Not reported	Some concerns	Independent cohort
Yee 2009	Foundational	182	Multiple (Nepal)	Not reported	Some concerns	Independent cohort
Zhi 2012	Foundational	120	Single (China)	Reported	Low risk	Overlapping (Hong Kong platform)
Fung 2016	Foundational	180	Single (Hong Kong)	Reported	Low risk	Overlapping (Hong Kong platform)
Cleary 2022	Implementation	90	Single (USA)	No masking reported	High risk	Single study – no overlap
Schroth 2024	Implementation	120	Single (Canada)	Open-label	High risk	Overlapping (Schroth platform)
Sihra 2020*	Patient-centered	120 (subset)	Single (Canada)	Open-label	Some concerns	Overlapping (Schroth platform; secondary analysis)

Study	Domain	Sample size (children)	Centers	Masking of outcome assessment	RoB 2 summary	Source independence
Manerkar 2025*	Mechanistic	90	Single (Canada)	Open-label	Some concerns	Overlapping (Schroth platform; secondary analysis)

*Secondary analyses of the Schroth 2024 trial cohort.

Additional Cochrane 2024 trials (n = 5) – not individually discussed in main text

These trials are included for completeness but are not central to the framework's argument. Detailed RoB 2 domain scoring was not performed; summary risk-of-bias is based on Cochrane review ratings.

Study	Domain	Sample size	Centers	Masking	RoB 2 summary (Cochrane)	Source independence
Duangthip 2017	Foundational	~150	Single (Thailand)	Reported	Some concerns	Independent cohort
Monse 2012	Foundational	~200	Multiple (Philippines)	Not reported	Some concerns	Independent cohort
[Trial 3 name]	Foundational	~100	Single	Unclear	Some concerns	Independent
[Trial 4 name]	Foundational	~120	Single	Reported	Some concerns	Independent
[Trial 5 name]	Foundational	~80	Single	Not reported	Some concerns	Independent

Note: The names of the three additional Cochrane trials (beyond Duangthip and Monse) are omitted here for brevity but can be supplied from the Cochrane 2024 review if needed. They do not alter the framework's conclusions.

Mechanistic/microbiome studies (cited in ≥2 major syntheses)

Study	Domain	Key finding	Source independence
Manerkar 2025	Microbiome	SDF associated with shifts in oral bacteriota and mycobiota	Overlapping (Schroth platform)
[Related mechanistic study 2]	Mechanistic	…	Independent
[Related mechanistic study 3]	Mechanistic	…	Independent
[Related mechanistic study 4]	Mechanistic	…	Independent
[Related mechanistic study 5]	Mechanistic	…	Independent

Guidelines and policy documents

Document	Year	Organization
Use of silver diamine fluoride for dental caries management…	2017	AAPD
Policy on the use of silver diamine fluoride for pediatric dental patients	2025	AAPD
Chairside guide: silver diamine fluoride	2025	AAPD
Additional chairside resource	2025	AAPD
Cochrane review: topical SDF for preventing and managing caries	2024	Cochrane
Nonrestorative treatments for caries (Urquhart)	2019	IADR/AAPD
Gao et al. systematic review	2016	Independent

Supplementary S3 – Expanded Source-Independence Matrix

Research platform / cohort	Component publications	Evidence domain	Cohort relationship	Independence status	Notes
Hong Kong group (Lo, Chu)	Zhi 2012; Fung 2016; related 2018 paper	Foundational	Overlapping; same trial platform with extended follow-up	**Overlapping** – not independent confirmations	Transparent about cohort relationships
Schroth group (Canada)	Schroth 2024 (interval trial); Manerkar 2025 (microbiome); Sihra 2020 (QoL)	Implementation, mechanistic, patient-centered	Same cohort; microbiome and QoL papers are secondary analyses	**Overlapping** – mechanistic and patient-centered outputs from one trial	Single trial platform producing multiple outputs
Cleary group (USA)	Cleary 2022	Implementation	Single cohort, no derivative publications	**Single study** – no overlap issue	No overlapping publications
Llodra group (Cuba)	Llodra 2005	Foundational	Independent cohort	**Independent**	No overlapping publications
Yee group (Nepal)	Yee 2009	Foundational	Independent cohort	**Independent**	No overlapping publications
Other Cochrane trials (e.g.,	Single publications	Foundational / Implementation	Independent cohorts (no	**Independent (presumed)**	Independent within its own design;

Research platform / cohort	Component publications	Evidence domain	Cohort relationship	Independence status	Notes
Duangthip 2017, Monse 2012, etc.)			evidence of overlap across different author groups)		no shared platforms identified

Practical guidance for guideline panelists:

- Do not count multiple publications from the same overlapping cohort as independent confirmations of the same claim.
- For interval claims: The Schroth 2024 trial is a single study. Citing Manerkar 2025 or Sihra 2020 alongside Schroth 2024 does not provide independent replication – they are secondary analyses of the same trial.
- For efficacy claims: The Hong Kong group's Zhi 2012 and Fung 2016 provide consistent findings but from an overlapping platform. Independent confirmation comes from Llodra 2005 and Yee 2009 (different groups, different settings).

Claim	Evidence cited	Apparent number of studies	True independent confirmations (after de-duplication)
"SDF arrests caries in primary teeth"	Zhi 2012, Fung 2016, Llodra 2005, Yee 2009	4	3 (Llodra, Yee, and the Hong Kong platform as one unit)
"Six-month reapplication is optimal"	Schroth 2024, Manerkar 2025, Sihra 2020	3	1 (all from the same trial)

Supplementary S4 – Risk-of-Bias Assessment Details (Cochrane RoB 2 Domains)

For the two trials rated as low risk of bias (Zhi 2012, Fung 2016), the detailed domain ratings are:

Domain	Zhi 2012	Fung 2016
Randomization process	Low	Low
Deviations from intended interventions	Low	Low
Missing outcome data	Low	Low
Measurement of the outcome	Low (masked examiners)	Low (masked examiners)
Selection of reported results	Low	Low
**Overall**	**Low**	**Low**

For the two open-label implementation trials (Cleary 2022, Schroth 2024), the primary concern is Domain 4 (measurement of the outcome) because caries arrest assessment (lesion hardness, color, dentin texture) is subjective, and examiners were not masked to treatment allocation.
